# First insights into using outline-based geometric morphometrics of wing cell contours to distinguish three morphologically similar species of *Tabanus* (Diptera: Tabanidae)

**DOI:** 10.1016/j.crpvbd.2024.100218

**Published:** 2024-10-03

**Authors:** Tanasak Changbunjong, Thekhawet Weluwanarak, Tanawat Chaiphongpachara

**Affiliations:** a Department of Pre-Clinic and Applied Animal Science, Faculty of Veterinary Science, Mahidol University, Nakhon Pathom, 73170, Thailand; b The Monitoring and Surveillance Center for Zoonotic Diseases in Wildlife and Exotic Animals (MoZWE), Faculty of Veterinary Science, Mahidol University, Nakhon Pathom, 73170, Thailand; c Department of Public Health and Health Promotion, College of Allied Health Sciences, Suan Sunandha Rajabhat University, Samut Songkhram, 75000, Thailand

**Keywords:** Geometric morphometrics, Horse flies, Species identification, Outlines, Vector, Wing cell

## Abstract

Accurate species identification of horse flies (Diptera: Tabanidae) is crucial due to their role as vectors for various pathogens, which is essential for understanding their biology, devising strategies to control their populations, and enhancing disease surveillance. This study assessed the efficacy of outline-based geometric morphometrics (GM) by analyzing the wing cell contours of discal, first submarginal, and second submarginal cells to distinguish three morphologically similar *Tabanus* species commonly found in Thailand, *T. megalops*, *T. rubidus*, and *T. striatus*. Statistical analysis demonstrated significant size differences between *T. rubidus* and the two other species (*P* < 0.05), with *T. rubidus* exhibiting larger wing cells. *Tabanus megalops* and *T. striatus* had similar sizes; their size differences were not statistically significant. The accuracy of size analysis based on validated classification tests was relatively low, ranging from 64.67% to 68.67%. Nonetheless, all wing cell contours showed significant shape differences between the three species, as confirmed by Mahalanobis distance comparisons using 1000 permutation tests (*P* < 0.05). The shape of the first submarginal cell contour showed the highest classification accuracy (86.67%). Outline-based GM offers a significant advantage for analyzing fly specimens with incomplete wings that have intact cells. For damaged specimens, analyzing the contour of the first submarginal cell through this technique can be a viable alternative.

## Highlights

• Wings of *Tabanus megalops*, *T. rubidus*, and *T. striatus* were examined using outline-based geometric morphometrics (GM).
• The first submarginal cell contour provided the most accurate classification.
• Outline-based GM is a promising alternative method for identifying horse fly species.

## Graphical abstract

**Figure:**
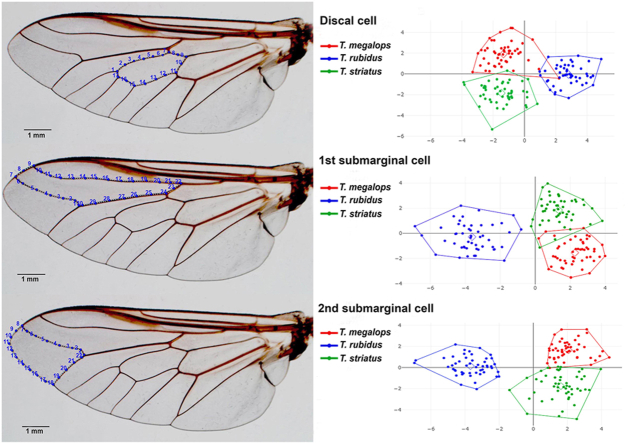

## 1 Introduction

Tabanid flies (Diptera: Tabanidae), encompassing over 4500 species across more than 144 genera, are a neglected group of hematophagous dipterans (Morita et al., 2016). The genus *Tabanus*, commonly known as horse flies, includes approximately 1300 species (Changbunjong et al., 2018b). These flies are important in the medicine and veterinary fields due to their blood-sucking behavior (Morita et al., 2016). Only female *Tabanus* flies feed on blood, primarily targeting domestic animals, livestock, wild animals, and occasionally humans. These flies act as mechanical vectors of animal *Trypanosoma* pathogens, such as *T. theileri*, *T. evansi*, and *T. vivax* (Baldacchino et al., 2014; Lendzele et al., 2022). They also spread pathogens that cause various infectious diseases, including African horse sickness, anthrax, bovine anaplasmosis, bovine besnoitiosis, bovine leucosis, equine infectious anemia, lumpy skin disease, and tularemia (Baldacchino et al., 2014). Additionally, horse flies inflict considerable economic losses on livestock production through irritation, stress, and blood loss, particularly impacting cattle and horses, leading to significant economic repercussions (Baldacchino et al., 2014). Understanding the biology of these fly vectors is crucial, but their identification remains challenging.

Accurate species identification of horse flies is crucial due to their role as vectors of various pathogens, which is essential for understanding their biology, devising strategies for controlling their populations in nature, and enhancing disease surveillance. The primary challenge in identifying these flies arises from their morphological similarity within certain groups, creating difficulties for taxonomists and frequently leading to misidentification using traditional morphology-based taxonomic identification methods (Changbunjong et al., 2018b). Such errors result in misconceptions when monitoring local horse fly species populations. *Tabanus megalops*, *Tabanus rubidus*, and *Tabanus striatus* are common in Thailand and exhibit similar morphological characteristics, which often lead to their misidentification (Changbunjong et al., 2021).

Molecular biology methods, such as DNA barcoding, are used to address the difficult problem of identifying these flies (Mugasa et al., 2018; Changbunjong et al., 2024). Recently, DNA barcoding techniques have been used in Thailand to identify horse flies. The results indicated that this technique can identify a wide range of fly species (Changbunjong et al., 2018a). However, some species, such as those within the *Tabanus ceylonicus* group, cannot be distinguished from one another (Changbunjong et al., 2018a). Furthermore, a major limitation of DNA barcoding is its high cost compared with that of standard morphological techniques. Therefore, other methods of horse fly identification should be explored, providing alternatives that can be appropriately used in different situations.

Geometric morphometrics (GM) is a modern technique that enhances the statistical analysis of shape variation through the configuration of landmarks on a target object rather than depending solely on measurements (Garros and Dujardin, 2013; Dujardin, 2017). This technique is notable for its low cost, as it only requires simple laboratory equipment, such as a basic microscope slide set, a microscope with a camera, and a computer (Dujardin, 2008, 2017). However, GM can pose challenges during dissection and sample preparation, which may be time-consuming, depending on the examined insect group or organ. The analysis after these stages is relatively quick (Dujardin, 2017). GM is particularly useful in situations where budget constraints limit project resources; it is a suitable alternative to be used in conjunction with traditional morphological methods for identifying horse fly species.

Insect wings are commonly used in GM to distinguish between morphologically similar insects because of their rigid two-dimensional structures, reducing digitizing errors (Dujardin, 2017; Lorenz et al., 2017). Furthermore, many fly species have unique wing shapes, which enhances the effectiveness of this method. Recently, GM has been used to identify various flies, including muscid flies (Limsopatham et al., 2021), horse flies (Changbunjong et al., 2021; Rodrigues et al., 2024), *Stomoxys* flies (Changbunjong et al., 2016), and blow flies (Limsopatham et al., 2022). These species were successfully identified using landmark-based GM, which relies on anatomical landmarks placed across the wing to generate shapes for analysis. However, this method needs a complete wing to be available for accurate analysis (Dujardin, 2017).

The wings of flies are reinforced by numerous longitudinal veins that often form interconnected closed “cells” in the membrane. Recent studies on the wing cells of various insect vectors, such as mosquitoes (Chonephetsarath et al., 2021; Laojun et al., 2024), biting midges (Hadj-Henni et al., 2023), and *Stomoxys* flies (Weluwanarak et al., 2024), highlight their unique characteristics suitable for species identification. However, no studies have been published on the differences in wing cells of horse flies that can be used to analyze interspecies variations. Using landmark-based GM to analyze these features presents challenges due to the difficulty of precisely locating anatomical landmarks (Dujardin et al., 2014). Outline-based GM, specifically designed to analyze the contours or boundary outlines of objects such as wing cells, is a viable alternative (Dujardin et al., 2014). The advantage of using wing cells for species classification through outline-based GM is that it enables the analysis of samples with incomplete wings as long as the wing cells remain intact.

In this study, we investigated the effectiveness of outline-based GM by analyzing the contours of three wing cells, the discal, first submarginal, and second submarginal cells, to distinguish between three morphologically similar species of *Tabanus* prevalent in Thailand, *T. megalops*, *T. rubidus*, and *T. striatus*. Additionally, we aimed to identify which wing cells exhibit the greatest interspecies differences, setting a target for future classification efforts of these flies. Our results are expected to provide an alternative method for identifying horse fly species, potentially enhancing the control and surveillance of their transmitted diseases.

## 2 Materials and methods

### 2.1 Horse fly collection and identification

*Tabanus megalops*, *T. rubidus*, and *T. striatus* were collected from November 2023 to February 2024 from seven provinces across five regions of Thailand: Chumphon (Southern Thailand); Prachuap Khiri Khan (Western Thailand); Phitsanulok, Uthai Thani, and Nakhon Pathom (Central Thailand); Nakhon Ratchasima (Northeastern Thailand); and Sa Kaeo (Eastern Thailand) (Table 1). The collection sites were chosen based on previous studies identifying these locations as habitats for these horse fly species (Changbunjong et al., 2018b, 2021). Five Nzi traps were strategically placed near beef cattle and buffalo farms and operated daily from 6:00 to 18:00 h over three consecutive days. All collected flies were euthanized by freezing at approximately −10 °C using a portable field freezer and then stored in 1.5 ml microcentrifuge tubes. The specimens were subsequently transported to the Vector-Borne Diseases Research Unit, Faculty of Veterinary Science, Mahidol University, Thailand, for further species identification.

**Table 1:** Collection sites and sample sizes (*n*) for *Tabanus megalops*, *Tabanus rubidus*, and *Tabanus striatus* for use in outline-based geometric morphometric analyses.

Species	Month/Year	Province (coordinates)	*n*
*T. megalops*	December 2023	Chumphon (10°29′33″N, 99°08′28″E)	20
	November 2023	Phitsanulok (16°49′41″N, 100°16′28″E)	15
	January 2024	Prachuap Khiri Khan (12°12′28″N, 100°00′25″E)	15
*T. rubidus*	December 2023	Chumphon (10°29′33″N, 99°08′28″E)	20
	November 2023	Uthai Thani (15°24′13″N, 100°00′49″E)	15
	February 2024	Nakhon Ratchasima (14°16′54″N, 102°28′16″E)	15
*T. striatus*	January 2024	Sa Kaeo (13°57′12″N, 102°21′05″E)	25
	January 2024	Nakhon Pathom (14°01′10″N, 99°57′37″E)	25
Total			150

*Note*: The three wing cell contours were evaluated using the same sample set.

All female horse fly specimens were morphologically identified using a stereomicroscope and taxonomic keys for *Tabanus* spp. in Thailand (Burton, 1978). Briefly, the morphological characteristics used to differentiate the three studied *Tabanus* species were as follows. Unlike *T. striatus*, *T. megalops* has a strip of pale tomentum and hairs across the midline of the second tergite. Additionally, the dark pattern on the abdominal dorsum of *T. striatus* is typically darker than that of *T. megalops*. *Tabanus rubidus* differs from these species in its basal callus, which is more triangular than rectangular (Burton, 1978; Changbunjong et al., 2021). Specimens with damaged morphological characteristics were excluded from the study. The identified specimens were stored in a freezer at −20 °C pending further analysis and wing slide preparation.

### 2.2 Wing image preparation

The specimens of *T. megalops*, *T. rubidus*, and *T. striatus*, which were morphologically identified and had intact left wings, were prepared for GM analysis; the numbers of specimens are detailed in Table 1. Wing slide preparation began with the careful dissection of the left wings from the bodies of the flies. Each wing was then placed on a slide, covered with a coverslip, and mounted using Hoyerʼs medium. The prepared slides were air-dried for one week. Subsequently, wing images were captured using a digital camera attached to a stereomicroscope (Nikon AZ 100, Nikon Corp., Tokyo, Japan), and a 1 mm scale-bar was included in each image for calibration.

### 2.3 Geometric morphometric (GM) analysis

**Fig. 1:**
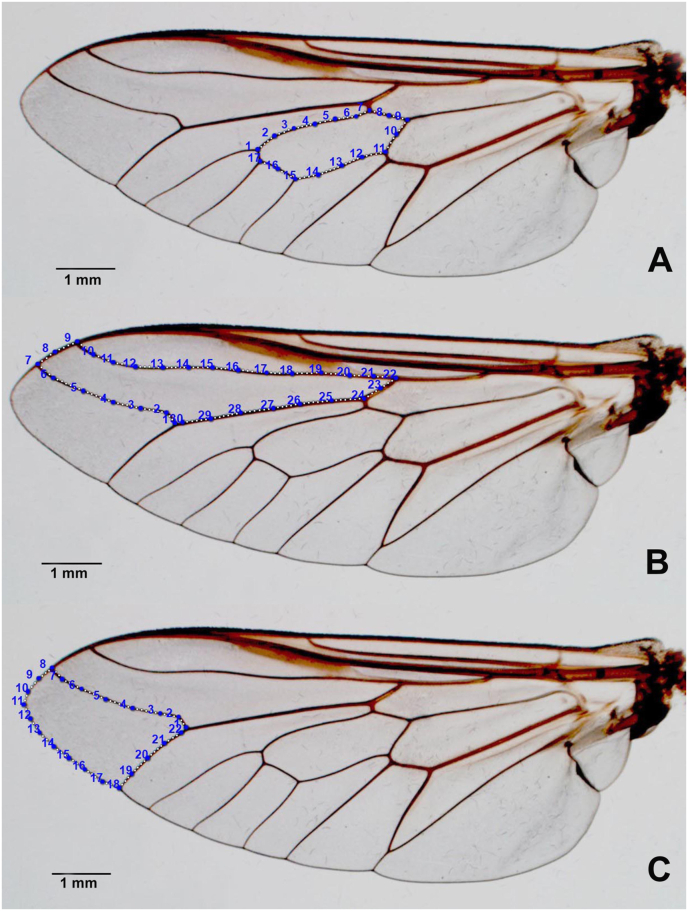
Wing cell contours of *Tabanus* spp. used for outline-based geometric morphometric analyses: discal cell (**A**), first submarginal cell (**B**), and second submarginal cell (**C**).

The outline-based GM analysis in this study focused on three wing cells, i.e. the discal, first submarginal, and second submarginal cells (Fig. 1). These wing cells were selected because they are large, polygonal cells with relatively thick membranes, which are difficult to damage compared to the thinner membranes of lower wing cells. After the wing cell contours were digitized, the coordinates were subjected to elliptic Fourier analysis (Kuhl and Giardina, 1982). The outlines were normalized by adjusting the coefficients for orientation and size using the semimajor axis of the first ellipse in the Fourier decomposition. Because the perimeter is easy to determine and is generally highly correlated with the semimajor axis, the perimeter of each wing cell contour was measured for comparative size analysis between *T. megalops*, *T. rubidus*, and *T. striatus*. The statistical significance of the perimeter differences between the species was determined using non-parametric permutation tests (1000 runs) with Bonferroni correction at a significance level of *P* < 0.05.

The shape of the wing cell contours was quantified using normalized elliptic Fourier coefficients. These coefficients were subsequently analyzed through principal component analysis to extract principal components (PCs). These PCs served as the final shape variables for discriminant analysis (DA), visualized on a factor map. Mahalanobis distances were then calculated to quantify the differences between species. The statistical significance of contour shape differences was assessed by comparing these distances using a non-parametric permutation test (1000 runs) with Bonferroni correction at a significance level of *P* < 0.05. To evaluate the effectiveness of each wing cell contour for species classification based on size and shape, we used two methods: the maximum likelihood method for assessing size (Dujardin et al., 2017) and the DA method based on the Mahalanobis distance for evaluating shape (Manly, 2004). Both methods were validated using the leave-one-out technique, as described by Manly (2004), where classification accuracy was calculated as a percentage. This technique was individually applied to each specimen, involving blinded reclassification of each, for a rigorous assessment of classification reliability. In total, 47, 42, and 41 PCs were used as inputs for the DA of the discal, first submarginal, and second submarginal cells, respectively.

The influence of contour size on contour shape, also known as the allometric effect, was assessed by calculating the coefficient of determination (*r*^2^). This was accomplished by regressing the first discriminant factor (DF) of each wing cell contour against the perimeter of that contour.

### 2.4 GM software

XYOM (XY online morphometrics) version 3 (Dujardin and Dujardin, 2019), an online application implementing the GM approach, was used for all analyses in this study. This online application is freely available on https://xyom.io/.

## 3 Results

In this study, 150 wings were analyzed using outline-based GM. These included 50 wings each from *T. megalops*, *T. rubidus*, and *T. striatus*. All wings were used in the analysis of the three wing cells.

### 3.1 Size analysis

**Fig. 2:**
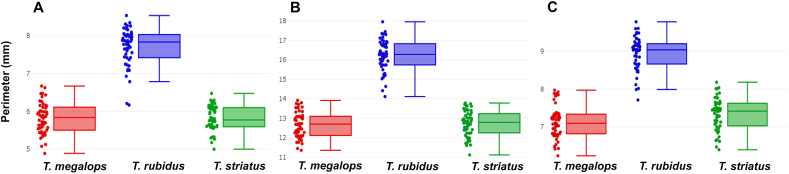
Boxplots illustrating wing cell perimeter variations between *Tabanus megalops* (*red*), *Tabanus rubidus* (*blue*), and *Tabanus striatus* (*green*): discal cell (**A**), first submarginal cell (**B**), and second submarginal cell (**C**). Dots represent the perimeter values for each specimen. The horizontal line within each box represents the median, separating the data into the 25th and 75th quartiles.

The size variations based on the perimeter of each wing cell contour are displayed in Fig. 2. The mean perimeters of the three wing cell contours varied, with measurements ranging from 5.81 mm for *T. striatus* to 7.70 mm for *T. rubidus* for the discal cell; from 12.68 mm for *T. megalops* to 16.23 mm for *T. rubidus* for the first submarginal cell; and from 7.12 mm for *T. megalops* to 8.95 mm for *T. rubidus* for the second submarginal cell (Table 2). Statistical analysis revealed significant contour size differences between *T. rubidus* and the two other species (*P* < 0.05), with *T. rubidus* having significantly larger wing cells. The contour size differences between *T. megalops* and *T. striatus* were not statistically significant, indicating that these species had similar wing sizes (*P* > 0.05; Table 2).

**Table 2:** Perimeter of wing cell contours of *Tabanus megalops*, *Tabanus rubidus*, and *Tabanus striatus* and statistically significant differences.

Species	*n*	Mean (mm)	Min-Max	Variance	SD
Discal cell					
*T. megalops*	50	5.82^A^	4.89–6.67	0.17	0.41
*T. rubidus*	50	7.70^B^	6.16–8.53	0.25	0.50
*T. striatus*	50	5.81^A^	5.00–6.47	0.11	0.33
First submarginal cell					
*T. megalops*	50	12.68^A^	11.35–13.91	0.43	0.66
*T. rubidus*	50	16.23^B^	14.11–17.95	0.66	0.81
*T. striatus*	50	12.72^A^	11.11–13.78	0.42	0.65
Second submarginal cell					
*T. megalops*	50	7.12^A^	6.24–7.97	0.19	0.44
*T. rubidus*	50	8.95^B^	7.71–9.77	0.19	0.44
*T. striatus*	50	7.33^A^	6.40–8.18	0.18	0.42

*Note*: The superscript letters following the mean perimeter values denote statistically significant differences at *P* < 0.05.

*Abbreviations*: *n*, sample size, Min, minimum; Max, maximum, SD, standard deviation.

### 3.2 Shape analysis

**Fig. 3:**
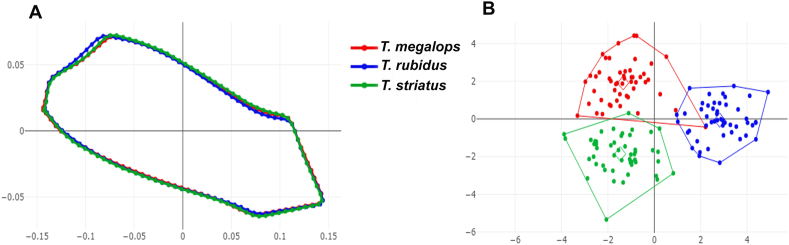
Shape variation of discal cell contour between *Tabanus megalops* (*red*), *Tabanus rubidus* (*blue*), and *Tabanus striatus* (*green*), as analyzed using outline-based geometric morphometrics. Superposition of aligned mean contour configurations (**A**) and factor map of the first two discriminant factors (DFs) of the three species (**B**). In the factor map, each point represents an individual from each species. The horizontal axis represents the first DF, accounting for 62.7% of the variance, and the vertical axis represents the second DF, accounting for 37.3% of the variance.

**Fig. 4:**
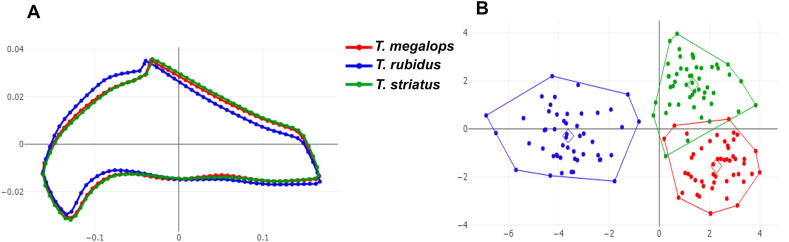
Shape variation of first submarginal cell contour between *Tabanus megalops* (*red*), *Tabanus rubidus* (*blue*), and *Tabanus striatus* (*green*), as analyzed using outline-based geometric morphometrics. Superposition of aligned mean contour configurations (**A**) and factor map of the first two discriminant factors (DFs) of the three species (**B**). In the factor map, each point represents an individual from each species. The horizontal axis represents the first DF, accounting for 77.1% of the variance, and the vertical axis represents the second DF, accounting for 22.9% of the variance.

**Fig. 5:**
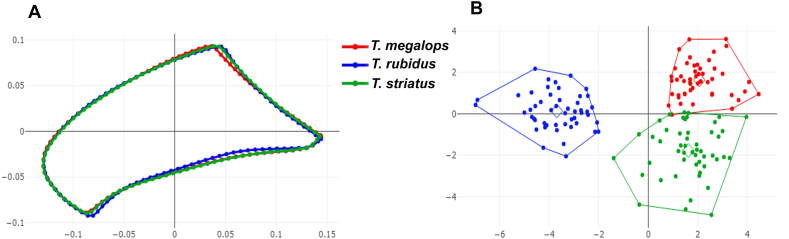
Shape variation of second submarginal cell contour between *Tabanus megalops* (*red*), *Tabanus rubidus* (*blue*), and *Tabanus striatus* (*green*), as analyzed using outline-based geometric morphometrics. Superposition of aligned mean contour configurations (**A**) and factor map of the first two discriminant factors (DFs) of the three species (**B**). In the factor map, each point represents an individual from each species. The horizontal axis represents the first DF, accounting for 78% of the variance, and the vertical axis represents the second DF, accounting for 22% of the variance.

The aligned mean wing cell contour configurations for the three fly species, *T. megalops*, *T. rubidus*, and *T. striatus*, are superposed in Fig. 3A (discal cell), Fig. 4A (first submarginal cell), and Fig. 5A (second submarginal cell). These graphics revealed contour shape differences between the three species for each wing cell.

The visualizations of the factor maps based on DA showed the shape variations of the three species in each wing cell contour, as displayed in Fig. 3B (discal cell), Fig. 4B (first submarginal cell), and Fig. 5B (second submarginal cell). DA revealed that two DFs explained 100% of the total variation in all three wing cell contours: DF1 accounted for 62.7% and DF2 for 37.3% for the discal cell; DF1 for 77.1% and DF2 for 22.9% for the first submarginal cell; and DF1 for 78% and DF2 for 22% for the second submarginal cell. The factor maps for the discal cell contour showed a minor overlap between the three species, whereas those for the first and second submarginal cells showed a slight overlap between *T. megalops* and *T. striatus*; *T. rubidus* was distinctly separated. Despite these overlaps, all wing cell contours significantly differed between the three species, as confirmed by the Mahalanobis distance comparisons, which were performed using a permutation test with 1000 runs (*P* < 0.001; Table 3).

**Table 3:** Pairwise Mahalanobis distances (below diagonal) for wing cell contours and their statistically significant differences after 1000 permutations (*P*-values; above diagonal) between *Tabanus megalops*, *Tabanus rubidus*, and *Tabanus striatus*.

Wing cell	Species	*T. megalops*	*T. rubidus*	*T. striatus*
Discal cell	*T. megalops*	–	<0.001	<0.001
	*T. rubidus*	4.60	–	<0.001
	*T. striatus*	3.77	4.65	–
First submarginal cell	*T. megalops*	–	<0.001	<0.001
	*T. rubidus*	6.02	–	<0.001
	*T. striatus*	3.52	5.40	–
Second submarginal cell	*T. megalops*	–	<0.001	<0.001
	*T. rubidus*	5.94	–	<0.001
	*T. striatus*	3.43	5.64	–

### 3.3 Validated classification

Each specimen was classified using validated classification based on size and shape to assess the accuracy of species classification using outline-based GM through analyses of the three wing cell contours. The shape of wing cell contours enabled better species classification than their size, as shown in Table 4. The highest total accuracy rate for species classification was observed for the first submarginal cell contour (86.67%), followed by the second submarginal cell contour (85.33%) and the discal cell contour (79.33%).

**Table 4:** Classification accuracy scores of validated classification based on the size and shape of wing cell contours of *Tabanus megalops*, *Tabanus rubidus*, and *Tabanus striatus*.

Species	Size		Shape	
	Assigned/Observed	Accuracy (%)	Assigned/Observed	Accuracy (%)
Discal cell				
*T. megalops*	47/50	94	37/50	74
*T. rubidus*	48/50	96	42/50	84
*T. striatus*	2/50	4	40/50	80
Total	97/150	64.67	119/150	79.33
First submarginal cell				
*T. megalops*	27/50	54	43/50	86
*T. rubidus*	46/50	92	46/50	92
*T. striatus*	25/50	50	41/50	82
Total	98/150	65.33	130/150	86.67
Second submarginal cell				
*T. megalops*	29/50	58	43/50	86
*T. rubidus*	45/50	90	49/50	98
*T. striatus*	29/50	58	36/50	72
Total	103/150	68.67	128/150	85.33

### 3.4 Allometric effects

To examine the allometric effects, we computed linear determination coefficients after regressing the contour shape DFs against the contour perimeter. The analysis revealed high coefficients for all three wing cell contours: 65.3% for the discal cell, 58.4% for the first submarginal cell, and 66.7% for the second submarginal cell. Thus, the contour size of the wing cells significantly influenced the variations in contour shape of the three *Tabanus* spp.

## 4 Discussion

Horse flies are significant veterinary and medical parasites, yet they remain understudied. A major challenge in studying these flies lies in their accurate identification, especially for morphologically similar species. In Thailand, *T*. *megalops*, *T. rubidus*, and *T. striatus* are common and often coexist in the same areas, complicating identification efforts (Changbunjong et al., 2018b). Although taxonomic manuals delineate the important morphological characteristics necessary for identifying these species, practical identification remains challenging because field specimens are often morphologically incomplete (Burton, 1978; Changbunjong et al., 2021). Therefore, alternative methods must be integrated with traditional morphological approaches to enhance identification accuracy.

GM offers a cost-effective alternative that requires only basic scientific equipment (Dujardin, 2008, 2017). Landmark-based GM was recently used in Thailand to classify *T. megalops*, *T. rubidus*, and *T. striatus*, achieving accuracy scores ranging from 94.38% to 99.39% (Changbunjong et al., 2021). Although highly efficient, this technique requires complete wing specimens (without tears), as it depends on anatomical landmarks distributed across the wing for precise analysis. To the best of our knowledge, this study is the first to demonstrate the effectiveness of using outline-based GM analysis on wing cells for classifying *Tabanus* species, providing a viable alternative for examining field specimens that have incomplete wings but intact wing cells.

Examination of the allometric effect revealed that the size (perimeter) of the three wing cells influences their shape. Typically, allometric effects should be excluded from analyses of intraspecific variations to prevent size variables from interfering with shape analyses (Dujardin, 2008, 2017). However, in studies on interspecific differences, the allometric effect is often retained because size and shape relationships can be crucial species-specific characteristics (Dujardin, 2008, 2017; Lorenz et al., 2017). Consequently, in this study, we preserved allometry in the final shape data to maintain the influence of species-specific traits. Our findings align with the relationships between wing size and shape observed in various fly species, such as *Stomoxys bengalensis*, *Stomoxys calcitrans*, and *Stomoxys sitiens* (Changbunjong et al., 2023), as well as *T. megalops*, *T. rubidus*, and *T. striatus* (Changbunjong et al., 2021). Therefore, although wing cells are only a subset of the wing, they are subject to similar influences as the entire wing.

Analysis of the size differences between the *T. rubidus*, *T. megalops*, and *T. striatus* wing cells consistently showed that *T. rubidus* has significantly larger wing cells than the two other species. This finding aligns with previous research that noted larger overall wing sizes in *T. rubidus* compared with *T. megalops* and *T. striatus* (Changbunjong et al., 2021). However, although the analysis indicated that *T. rubidus* has larger wing cells, determining their usefulness for species classification remains challenging. The use of wing size as a classification variable can be inaccurate because insect wing size is sensitive to environmental conditions (Jirakanjanakit et al., 2007; Morales Vargas et al., 2010; Baleba et al., 2019). Factors such as larval density and various substrates, including camel, cow, donkey, and sheep dung, during the larval stages of *S. calcitrans* directly influence wing size (Baleba et al., 2019). Because wing cell contour size correlates with the overall wing size, it is equally susceptible to environmental conditions. Therefore, wing cell contour size should not be the primary criterion for species identification decisions. Furthermore, the accuracy of size analysis based on validated classification for species classification is relatively low, ranging from 64.67% for the discal cell to 68.67% for the second submarginal cell, primarily due to the lack of size differences between *T. megalops* and *T. striatus*.

Shape variation, as analyzed through GM, is a robust approach to species classification (Limsopatham et al., 2022). Shape attributes contain vital information about genetic variations, which is essential for distinguishing species (Dujardin, 2008, 2017). Unlike size, shape is considerably less influenced by environmental factors, especially in analyses of interspecific differences (Chonephetsarath et al., 2021). Statistical comparisons revealed that the three horse fly species exhibited distinct wing cell contours across all analyses. Although statistical differences were observed across all species in all wing cell contours, further analysis showed that the contours have varying degrees of discriminatory power. Superposition of the aligned mean wing cell contour configurations indicated that the first submarginal cell contour displays the most significant differences among the three species. Additionally, the DA-based factor maps indicated that in the first and second submarginal cells, *T. rubidus* diverges into distinct groups without overlapping with the other species. These variations affected the classification accuracies for each wing cell, with the first submarginal cell contour achieving the highest accuracy (86.67%), followed by the second submarginal cell (85.33%) and the discal cell (79.33%), according to validated classification. The primary reason for the higher classification accuracy for the first submarginal cell contour may be its longitudinal nature; it spans more than half the wing length, possibly capturing the unique characteristics of each fly species.

Although no studies have specifically investigated wing cell contour shape differences in horse flies, research on mosquitoes shows that different wing cell contours yield varying levels of species identification accuracy, depending on the tested mosquito species (Chonephetsarath et al., 2021; Laojun et al., 2024). The similarity patterns between the wing cell contours of *T. megalops*, *T. rubidus*, and *T. striatus* align with previous findings on species-specific wing shapes. This alignment confirms that wing cell contour shape characteristics are consistent with the whole wing shape (Changbunjong et al., 2021).

Our study indicates that the first submarginal cell contour is the most accurate for species classification among the wing cells analyzed, with a satisfactory overall accuracy of 86.67%. However, outline-based GM using wing cell contour analyses are less accurate than the landmark-based GM used in a previous study (Changbunjong et al., 2021), where anatomical landmarks across the entire wing were analyzed (86.67 *vs* 96.59%). This is expected, as whole-wing analysis provides more species-specific information than analyses based solely on wing cells, which are merely subsets of wings. Although landmark-based GM should be the primary approach to species classification, the present study suggests that the contour of the first submarginal cell can be analyzed using outline-based GM as a viable alternative in cases where specimens have damaged wings and missing landmarks.

## 5 Conclusions

This study is the first to demonstrate the effectiveness of using outline-based GM on wing cell contours to classify three morphologically similar *Tabanus* species, *T. megalops*, *T. rubidus*, and *T. striatus*. This approach is a viable alternative for analyzing field specimens that have incomplete wings but intact wing cells. The contour of the first submarginal cell enables the most accurate classification, achieving a satisfactory overall accuracy rate. Future research should further explore the classification efficiency of this method for other *Tabanus* spp. The results of this study provide an alternative method for identifying horse fly species, potentially enhancing the control and surveillance of their transmitted diseases.

## Funding

This research was funded by 10.13039/501100004156Mahidol University (Fundamental Fund: fiscal year 2024 by National Science Research and Innovation Fund (NSRF)), grant number FF-132/2567.

## Ethical approval

The study received approval from the Animal Care and Use Committee of the Faculty of Veterinary Science at Mahidol University, Thailand (Ref. MUVS-2023-10-70).

## Data availability

The data supporting the conclusions of this article are included within the article.

## CRediT authorship contribution statement

**Tanasak Changbunjong:** Conceptualization, Investigation, Methodology, Formal analysis, Data curation, Writing – original draft, Writing – review & editing, Visualization, Supervision, Project administration, Funding acquisition. **Thekhawet Weluwanarak:** Investigation, Methodology, Visualization, Formal analysis. **Tanawat Chaiphongpachara:** Conceptualization, Investigation, Methodology, Formal analysis, Data curation, Writing – original draft, Writing – review & editing, Visualization.

## Declaration of competing interests

The authors declare that they have no known competing financial interests or personal relationships that could have appeared to influence the work reported in this paper.
